# The Donor-Dependent and Colon-Region-Dependent Metabolism of (+)-Catechin by Colonic Microbiota in the Simulator of the Human Intestinal Microbial Ecosystem

**DOI:** 10.3390/molecules27010073

**Published:** 2021-12-23

**Authors:** Qiqiong Li, Florence Van Herreweghen, Marjan De Mey, Geert Goeminne, Tom Van de Wiele

**Affiliations:** 1 Center for Microbial Ecology and Technology (CMET), Department of Biotechnology, Faculty of Bioscience Engineering, Ghent University, Coupure Links 653, 9000 Ghent, Belgium; Qiqiong.Li@UGent.be (Q.L.); Florence.VanHerreweghen@UGent.be (F.V.H.); 2 Centre for Synthetic Biology (CSB), Department of Biotechnology, Faculty of Bioscience Engineering, Ghent University, Coupure Links 653, 9000 Ghent, Belgium; Marjan.DeMey@UGent.be; 3 VIB Metabolomics Core Ghent, VIB, 9052 Ghent, Belgium; Geert.Goeminne@psb.vib-ugent.be

**Keywords:** catechins, metabolites, identification and quantification, metabolic pathway, gut microbiota, SHIME system

## Abstract

The intestinal absorption of dietary catechins is quite low, resulting in most of them being metabolized by gut microbiota in the colon. It has been hypothesized that microbiota-derived metabolites may be partly responsible for the association between catechin consumption and beneficial cardiometabolic effects. Given the profound differences in gut microbiota composition and microbial load between individuals and across different colon regions, this study examined how microbial (+)-catechin metabolite profiles differ between colon regions and individuals. Batch exploration of the interindividual variability in (+)-catechin microbial metabolism resulted in a stratification based on metabolic efficiency: from the 12 tested donor microbiota, we identified a fast- and a slow-converting microbiota that was subsequently inoculated to SHIME, a dynamic model of the human gut. Monitoring of microbial (+)-catechin metabolites from proximal and distal colon compartments with UHPLC-MS and UPLC-IMS-Q-TOF-MS revealed profound donor-dependent and colon-region-dependent metabolite profiles with 5-(3′,4′-dihydroxyphenyl)-γ-valerolactone being the largest contributor to differences between the fast- and slow-converting microbiota and the distal colon being a more important region for (+)-catechin metabolism than the proximal colon. Our findings may contribute to further understanding the role of the gut microbiota as a determinant of interindividual variation in pharmacokinetics upon (+)-catechin ingestion.

## 1. Introduction

Catechins, members of the flavan-3-ol polyphenol family, are widely distributed in a range of dietary sources such as cocoa products, tea, berries and other fruits, including kiwi and apples [1,2]. Catechins possess various biological functions, including anti-inflammatory, antioxidative, antimicrobial, immunomodulatory, antimicrobial and neuroprotective effects [3]. Although there are a large number of studies supporting the various biological activities of catechins, the reported health outcomes are not always consistent. A key factor leading to these inconsistencies is the variability in the bioavailability and bioactivity among different study populations [4].

The in vivo bioavailability of catechins is extremely poor (around 1.68% in humans) [5]. A small fraction of the ingested catechins is absorbed and undergoes phase I and phase II metabolism within small intestinal enterocytes and liver by enzymes, such as glucuronosyltransferases, sulfotransferases and catechol-O-methyltransferase [6]. Approximately two-thirds of catechins reach the colon where catechins are degraded by microbial enzymes into a range of metabolites [7,8]. The human gastrointestinal tract is colonized by an extremely dense and diverse microbial community [9]. The enormous gene pool of the gut microbiota makes the colon a bioreactor with great potential for polyphenol metabolism [10]. The gut microbiota has the metabolic capacity to perform glycosidic linkages, C-ring fission and the degradation of the heterocyclic structures of catechins, forming smaller molecules, including phenylvalerolactones and phenylvaleric acids [8]. These generated microbial metabolites of a wide variety of new chemical structures are available for absorption across the colon epithelium and can eventually enter the systemic blood circulation [11]. The bioconversion of compounds is generally considered as a procedure inactivating biological activity and fostering their excretion [6]. However, in the case of catechins, the resulting metabolites from microbial biotransformation can contribute to the biological activity of catechins or are even more active than the parent compounds. For example, dihydroxy-γ-valerolactone derivatives, such as 5-(3′,4′-dihydroxyphenyl)-γ-valerolactone have been found to pathogenic bacteria in vitro [12]. Furthermore, C-ring cleavage metabolite of (epi)catechin, 1-(3′,4′-dihydroxyphenyl)-3-(2′′,4′′,6′′-trihydroxyphenyl)propan-2-ol exhibited stronger antioxidant capacity than that of parent compounds in DPPH radical-scavenging and ferric-reducing activity tests [13].

Given the important role of gut microbiota in the metabolism of catechins, the interindividual differences in gut microbiota composition may lead to different metabolic end products with various beneficial effects in vivo [14]. Considering the progressive increase of bacterial density from the proximal to distal colon within the same individual, gradients of bacterial catechin metabolites may exist in the longitudinal direction of the colon: this has for instance been demonstrated for the metabolism of catechin dimers (procyanidins) in rats [15]. However, studies so far rarely make a distinction between the metabolism profiles of catechins in different colon regions when studying human-microbiota-derived metabolites and their biological activity. This study aims to perform a direct comparison between two stratified donors and different colon regions concerning their microbial metabolites profile by a regular supplementation of (+)-catechin to the Simulator of the Human Intestinal Microbial Ecosystem (SHIME). This study provides evidence for donor-dependent and colon-region-dependent metabolism of (+)-catechin by gut microbiota.

## 2. Results

### 2.1. Interindividual Differences in Metabolic Rate of (+)-Catechin

It was hypothesized that the gut microbiota from different individuals metabolizes catechins, especially (+)-catechin in this study, with different metabolic efficiency. This was addressed by separately incubating (+)-catechin at 200 mg/L with the fecal microbiota derived from 12 different healthy human donors for 24 h. The (+)-catechin was qualified and quantified by UHPLC-MS analysis. At 24 h, there was no (+)-catechin detected in any of the samples, showing that supplemented (+)-catechin was rapidly and completely metabolized by the fecal microbiota from these 12 individuals. Our findings are supported by a recent study on four primary green tea catechins, demonstrating the short survival time of catechins in in vitro incubation with human fecal microbiota [16]. This is also in line with a clinical ^14^C-epicatechin absorption, distribution, metabolism and excretion (ADME) trial that showed the majority of ingested epicatechin was excreted within the first 24 h post-epicatechin intake [17]. We therefore assessed individual differences in metabolism efficiency defined as the conversion percentage of (+)-catechin at the time point of 4 h where the residual (+)-catechin was still detectable (Table 1). Fecal microbiota from Donors 1, 7 and 12 rapidly degraded over 40% (+)-catechin, and Donors 2, 6, 8 and 9 can be grouped as slow-converting microbiota due to almost no change in (+)-catechin concentration at 4 h. A similar result was retrieved upon assessing the metabolism of (−)-epicatechin with human fecal slurries, in which individuals were classified as slow and fast (−)-epicatechin metabolizers based on the conversion rate within the first 2 h of in vitro incubation [18]. The profound difference in metabolic conversion percentage of (+)-catechin after 4 h of incubation resulted in the stratification of fast and slow catechin-converting microbiota, respectively. Donor 1 and Donor 2 with personal availability and without medication history were enrolled for the subsequent SHIME run. Previous studies established the correlation of different microbial compositions with metabolites of catechins [18,19]. Thus, the microbial profiles of the fecal inoculum of these two selected donors were analyzed as shown in Supplementary Materials Figure S1. It was indicated that there were differences in composition and abundance at both phylum and genus levels between donors. As this only concerns two individuals, no statements on microbiome composition and metabolic efficiency can be made.

### 2.2. Identification of (+)-Catechin Metabolites during Simulated Colon Digestion in SHIME

**Figure 1:**
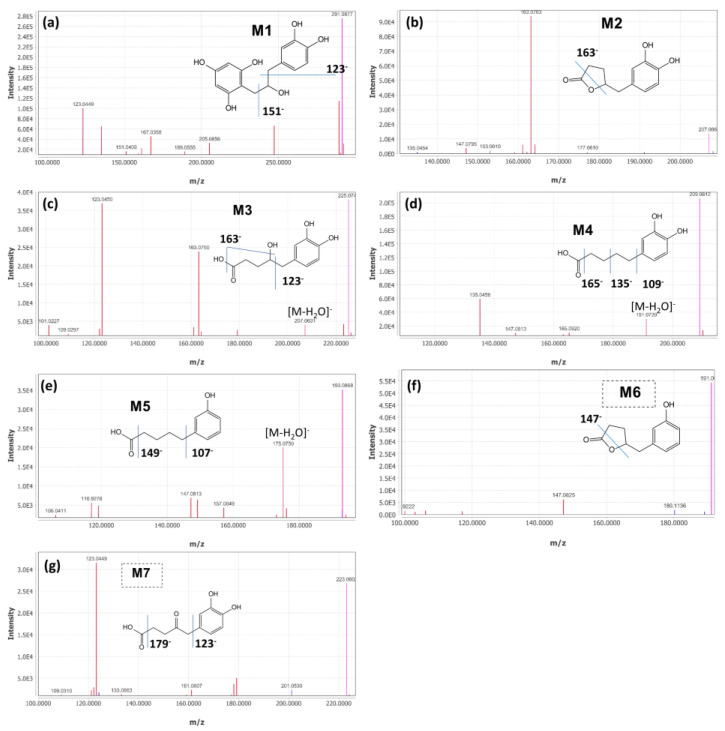
Mass fragment profiles of secondary ion fragments (MS^2^) of metabolites from UPLC-IMS-Q-TOF-MS. (**a**), M1, 1-(3′,4′-dihydroxyphenyl)-3-(2′′,4′′,6′′′-trihydroxyphenyl) propan-2-ol; (**b**), M2, 5-(3′,4′-dihydroxyphenyl)-γ-valerolactone; (**c**), M3, 4-hydroxy-(3′,4′-dihydroxyphenyl)-valeric acid; (**d**), M4, 5-(3′,4′-dihydroxyphenyl)-valeric acid; (**e**), M5, 5-(3′-hydroxyphenyl)-valeric acid; (**f**), M6, 5-(3′-hydroxyphenyl)-γ-valerolactone; (**g**), M7, 5-(3′,4′-dihydroxyphenyl)-4-oxo-valeric acid. The characteristic peaks contributing to the identification are labeled with their potential corresponding fragmentation pathways.

**Figure 2:**
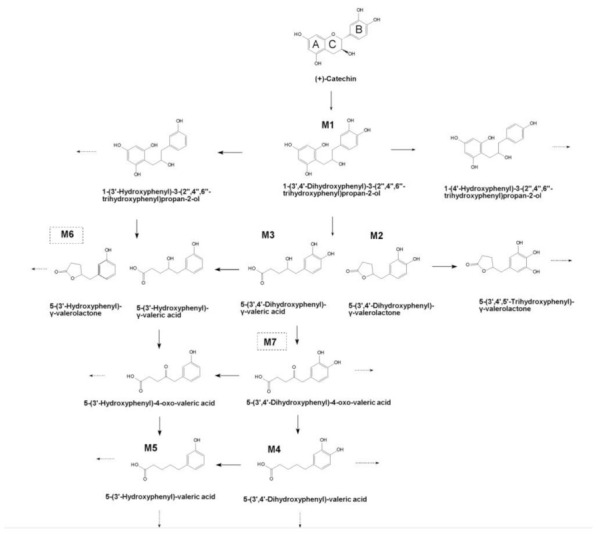
Potential microbial metabolism pathway of (+)-catechin by colonic microbiota in SHIME incubation. M1 to M7 were detected in the present study. Chemicals in boxes with dashed lines (M6 and M7) were detected by UPLC-IMS-Q-TOF-MS, but there were no quantitative data provided by UHPLC-MS analysis.

The fecal microbiota of fast-converting Donor 1 and slow-converting Donor 2 were inoculated in SHIME. Upon a 7-day stabilization period, which resulted in a proximal and distal colon microbiota in the respective SHIME runs, the SHIME reactors were supplemented with 3 × 200 mg/d (+)-catechin for 21 days. Characterization of catechin metabolism profiles in this study was monitored at the end of this treatment period. Based on the chromatography of authentic standards and mass fragment profiles in samples of (+)-catechin and control, (+)-catechin and its potential microbial metabolites were qualified and quantified. The analytical features of UPLC-IMS-Q-TOF-MS are summarized in Figure 1 and Table 2. In total, there were seven metabolites identified in this study. The putative chemical structures of these metabolites are provided in Figure 2.

As for (+)-catechin and M2, the mass measurements of authentic standards ensure accurate annotation. The accurate deprotonated molecule of M1 was at *m*/*z* 291.0877 [M-H]^−^ based on UPLC-IMS-Q-TOF MS data. Its molecular weight was also confirmed by UHPLC-MS analysis in positive and negative mode indicating protonated and deprotonated molecules at *m*/*z* 293 and 291, respectively. The product-ion spectrum for *m*/*z* 291 provided characteristic fragmentation ions at *m*/*z* 123, 247, 135 and 167, which is in agreement with previous reports [20,21]. The MS^2^ fragmentation at 123.04 and 135.04 originated from the loss of C_8_H_9_O_4_ and the combined loss of C_8_H_9_O_4_ and H_2_O from the parent ion, respectively. Based on present and published data, M1 is tentatively identified as 1-(3′,4′-dihydroxyphenyl)-3-(2′′,4′′,6′′-trihydroxyphenyl)-2-propanol. Similarly, we deduce M3 to be 4-hydroxy-(3′,4′-dihydroxyphenyl)-valeric acid, based on the deprotonated molecule at *m*/*z* 226 [M-H]^−^ and three characteristic fragment ions of 123.04, 163.07 and 207.06, which is in line with a previous study [22]. Likewise, M4 (*m*/*z* 210 [M-H]^−^), M5 (*m*/*z* 194 [M-H]^−^), M6 (*m*/*z* 192 [M-H]^−^) and M7 (*m*/*z* 224 [M-H]^−^) detected during the 120 h of incubation were indicated as 5-(3′,4′-dihydroxyphenyl)-valeric acid, 5-(3′-hydroxyphenyl)-valeric acid, 5-(3′-hydroxyphenyl)-γ-valerolactone and 5-(3′,4′-dihydroxyphenyl)-4-oxo-valeric acid, respectively, based on the corresponding fragmentation pathway and literature information [18,22,23]. For example, fragment ions at *m*/*z* 207.06, 191.07 and 175.07 of the valeric acid M3, M4 and M5 may be formed by the neutral loss of H_2_O. In this study, phenylvalerolctones (two metabolites) and phenolic acids (five metabolites) were the main identified products formed by colonic microbiota, which have been well documented as the major colonic metabolites and vital compounds in beneficial effects of flavan-3-ols [24].

Based on the obtained results and literature [25], we propose a tentative metabolic pathway of (+)-catechin by colonic microbiota in the SHIME system (Figure 2). The initial step of metabolism consists of the reductive opening of heterocyclic C-ring to yield M1 1-(3′,4′-dihydroxyphenyl)-3-(2′′,4′′,6′′-trihydroxyphenyl)-2-propanol. It is known that the C-ring cleavage is a critical metabolic step for the human microbial metabolism of several flavonoids [26]. In the case of catechins, this step facilitates the formation of diphenylpropanol (M1) and can be performed by several bacteria such as *Eggerthella lenta* and *Adlercreutzia equolifaciens* [8]. The resulting M1 then underwent the degradation of the phloroglucinol moiety to produce M2 5-(3′,4′-dihydroxyphenyl)-γ-valerolactone and M3 4-hydroxy-(3′,4′-dihydroxyphenyl)-valeric acid, which may be subsequently converted to corresponding valerolactone M6 and valeric acid by dephydroxylation at the 4′ position. *Eggerthella lenta* CAT-1 has the ability to dehydroxylate the B-ring of M1, leading to the formation of 1-(3′-hydroxyphenyl)-3-(2′′,4′′,6′′-trihydroxyphenyl)propan-2-ol [27]. Furthermore, M3 was biotransformed to M7 5-(3′,4′-dihydroxyphenyl)-4-oxo-valeric acid by oxidation of the 4-hydroxy group. After that, M4 5-(3′,4′-dihydroxyphenyl)-valeric acid was produced by reductive elimination of oxygen from M7, followed by the dehydroxylation at the 4′ position to form M5 5-(3′-hydroxyphenyl)-valeric acid. The formation of M5 can also be derived from 4′-dehydroxylated M3. It is noteworthy that M4 and M5 are not the end products of microbial metabolism of (+)-catechin, and they can be further converted by β-or α-oxidation [25]. The entire metabolic pathway of catechin may require the cooperation of different bacteria, but currently, only a few bacteria have been identified to be able to metabolize (+)-catechin [8]. To conclude, the formation of upstream metabolites involves the metabolism steps of C-ring opening, A-ring fission while the formation of downstream metabolites involves degradation reactions, including the dehydroxylation of the phenyl moiety and the shortening of the aliphatic chain of phenylvaleric acids.

### 2.3. Dynamics of (+)-Catechin Metabolism in SHIME Is Colon-Region- and Donor-Dependent

**Figure 3:**
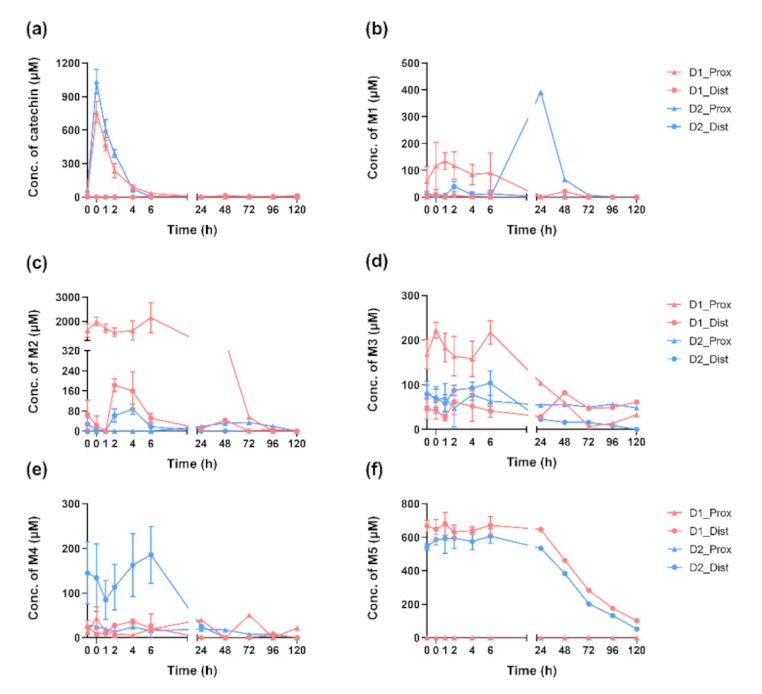
Colon-region- and donor-dependent quantitative analysis of (+)-catechin (**a**) and its microbiota-derived metabolites during 120 h incubation: (**b**) M1, 1-(3′,4′-dihydroxyphenyl)-3-(2′′,4′′,6′′-trihydroxyphenyl)propan-2-ol; (**c**), M2, 5-(3′,4′-dihydroxyphenyl)-γ-valerolactone; (**d**) M3, 4-hydroxy-(3′,4′-dihydroxyphenyl)-valeric acid; (**e**) M4, 5-(3′,4′-dihydroxyphenyl)-valeric acid; (**f**) M5, 5-(3′-hydroxyphenyl)-valeric acid. The concentration of (+)-catechin and metabolites from 0 h to 6 h are shown as mean ± SD from three sampling days. D1_Prox (pink, triangle), proximal colon vessel of donor 1; D1_Dist (pink, circle), distal colon vessels of donor 1; D2_Prox (blue, triangle), proximal colon vessel of donor 2; D2_Dist (blue, circle), distal colon vessels of donor 2.

The biotransformation of (+)-catechin by the colonic microbiota derived from fecal materials of two healthy donors was firstly determined by UHPLC-MS. The quantitative changes of the (+)-catechin and each microbial metabolite through incubation time (0, 0, 1, 2, 4, 6, 24, 48, 72, 96 and 120 h) are shown in Figure 3 and Supplementary Materials Figure S2. There were two 0 h sampling time points because samples were taken immediately before and after (+)-catechin supplementation in case of residual (+)-catechin from the previous supplementation. Notably, (+)-catechin was rapidly metabolized by the proximal colonic microbiota from both donors. After 6 h of incubation, a limited amount of (+)-catechin was detected in samples from the proximal colon vessel of both donors (less than 7% and 0.4% of the supplemented amount, respectively). In contrast to the huge interindividual variation in the conversion percentage of (+)-catechin in batch incubation, the level of residual (+)-catechin in incubation with SHIME colonic microbiota of both donors was rather low (50–100 µM) at 4 h. These differences in conversion percentage may be due to the additional stabilization period in SHIME to accommodate the presence of (+)-catechin and the different incubation conditions of these two in vitro techniques. Within 24 h, (+)-catechin was completely degraded in the proximal colon. The short residence time of (+)-catechin in the colon is in agreement with a previous study that found the 95% added (+)-catechin was degraded within 8 h in vitro incubation with rat fecal microbiota [13]. There was no (+)-catechin measured in the distal colon compartment during the 120 h incubation period. This indicates that the primary metabolic degradation of (+)-catechin in the human colon may mainly occur in the proximal colon as proposed for carbohydrates [28].

The concentration of microbial metabolites present in samples of treatment vessels but absent in control vessels was monitored by UHPLC-MS analysis. Despite being identified by UPLC-IMS-Q-TOF-MS analysis, there were no quantitative data for M6 and M7 due to the limits of quantification and the resolution of UHPLC-MS analysis. During the first 6 h of incubation, the types and amounts of metabolites produced by proximal and distal colonic microbiota of both donors did not show remarkable changes, whereas the level of (+)-catechin gradually decreased. It indicates that the microbial community in the SHIME colon compartments developed an enduring metabolic capacity for (+)-catechin and was able to maintain the (+)-catechin metabolites at a certain level. This stable metabolic profile may be attributed to the regular administration of (+)-catechin in the stabilization and treatment period, which allowed colonic microbiota to evolve in this semicontinuous dynamic.

Data from Supplementary Materials Figure S2 indicate that metabolic profiles of (+)-catechin in SHIME incubation colonic microbiota of two donors were different in terms of both types and levels of microbial (+)-catechin metabolites. For instance, M2 was the most abundant metabolite in the proximal colon of Donor 1 (fast converter), while M2 was not detectable in the proximal colon of Donor 2 (slow converter) in the first 6 h. In the meantime, mainly due to the high baseline of M2, the total concentration of all microbial metabolites of (+)-catechin in the proximal colon of Donor 1 was approximately 21 times higher than that of Donor 2 at 6 h. Similar to our results, M2 was also considered as a major contributor to the interindividual variability in the metabolism of flavan-3-ols in the previous human or in vitro research [29,30]. The donor-dependent metabolic profiles of (+)-catechin are in accordance with findings from the latest in vitro studies of other catechins, including (−)-epicatechin [18] and epigallocatechin gallate [16]. However, the differences in metabolic profiles in the distal colon of the two donors were much less notable. M5 was the major metabolite for both donors during 120 h incubation. These results suggested that the proximal colon might be the main region where interindividual variability in microbial metabolism of (+)-catechin originates. It should be noted that most (+)-catechin has been degraded into smaller molecules in the proximal colon and were subsequently transported to the distal colon. Therefore, the potential explanation of similar metabolic patterns in distal colons between individuals could be that the generated upstream metabolites are prone to be generally degraded by multiple bacteria than (+)-catechin.

Data from Figure 3 and Supplementary Materials Figure S2 reveal that the metabolite patterns obtained in the same donor, but different colon regions differed hugely. For Donor 1, the upstream metabolite M2 was the major microbial product in the proximal colon during incubation, while downstream metabolite M5 was dominant in the distal colon. Opposite to the other identified phenolic acid metabolites, phenyl-γ-valerolactones displayed a high level through the treatment period after regular supplementation of (+)-catechin, which was also highlighted in previous studies on flavan-3-ols [31,32]. Additionally, the types and concentrations of metabolites also varied greatly between colon regions. For instance, the mean amount of M2, M3 and M4 between regions during 6 h incubation differed up to 22.1-fold, 4.1-fold and 6.5-fold, respectively. As reported previously, the phenolic acids and small aromatics such as M4 and M5 (products of downstream metabolites) were mainly detected in the distal colon [15]. Thus, it can be concluded that distinct metabolic profiles of (+)-catechin occur in various colon regions. This is consistent with earlier findings, which demonstrated in vivo progressive nature of microbial metabolism in various regions of the colon from male Wistar rats [15]. In this study with simulated human colon microbiota, all seven identified compounds except for M1 were phenyl-γ-valerolactones and hydroxy-phenylvaleric acids, which were also reported to be major contributors to the bioavailability of catechins [33]. These results also confirm the high production of phenyl-γ-valerolactones and phenylvaleric acids as the main microbial metabolites of catechins as previously reported [33,34]. As expected, metabolites in the proximal and distal colons gradually declined during the washout period of 24 h to 120 h.

## 3. Materials and Methods

### 3.1. Chemicals

(+)-Catechin for incubation and standard of 5-(3′,4′-dihydroxyphenyl)-γ-valerolactone were obtained from Toronto Research Chemical (Toronto, Canada). Standards of (+)-catechin and HPLC-grade methanol were ordered from Carl Roth (Karlsruhe, Germany). HPLC-grade formic acid was purchased from Merck (Merck, Overijse, Belgium). Water for ultra-high-performance liquid chromatography–mass spectrometry (UHPLC-MS) was purified by a Milli-Q water purification system (Merck Millipore, Overijse, Belgium).

### 3.2. Batch Incubation

To evaluate the interindividual variability in conversion percentage of (+)-catechin, fecal materials were obtained from 12 healthy donors (5 males and 7 females, age 25–30 years) without dietary restrictions. None of the donors had antibiotics treatments or any gastrointestinal disease for at least 3 months prior to fecal donation. Experimental work with fecal material donated by human was approved by the ethical committee of Ghent University under the registration number B670201836318.

The collection of fecal samples and preparation of 20% (*w*/*v*) fecal slurries complied with the description by De Paepe et al. [35]. In short, fresh fecal samples were donated into airtight containers with AnaeroGen™ sachets (Oxoid Ltd., Basingstoke, Hampshire, UK) for eliminating O_2_. Then, 20 g of fecal material was added into 100 mL of anaerobic prereduced phosphate buffer saline (PBS, 0.1 M, pH 6.8) supplemented with 1 g/L sodium thioglycolate (Sigma Aldrich, Darmstadt, Germany) as a reducing agent. The fecal slurry was obtained after being homogenized in a stomacher (LabBlender 400, Seward Ltd., Worthing, West Sussex, UK) for 10 min, followed by centrifugation at 3000× *g* for 5 min. Before inoculation, the fecal slurries were washed once with prereduced PBS by centrifugation (3000× *g*, 10 min) and resuspension in PBS to remove potential residual (+)-catechin from feces. The resulting fecal supernatant was inoculated into penicillin bottles containing 44 mL of prereduced low-sugar medium and 1 mL of (+)-catechin to obtain a final concentration of 1% (*w*/*v*) feces and 200 mg/L (+)-catechin.

### 3.3. Experimental Design of SHIME

The Simulator of the Human Intestinal Microbial Ecosystem (SHIME^®^) was used to monitor the metabolism of (+)-catechin by the gut microbial community. The SHIME system is a semicontinuous multicompartment simulator of the human gut [36]. In this study, the SHIME system consisting of a double-jacketed combined with stomach and the small intestine vessel was adapted to run in parallel for two donors, each of which was equipped with two proximal colon vessels and two distal colon vessels (one set for (+)-catechin treatment and the other set for Milli-Q water as control). The schematic overview of this SHIME system is shown in Supplementary Materials Figure S3. The colon vessels were characterized at pH 5.6 to 5.9 (proximal) and 6.6 to 6.9 (distal) by the equipped pH controllers and electrodes. During the experimental period, all vessels were maintained at 37 °C by connecting to a warm water bath (Julabo, Seelbach, Germany) and mixed at 200 rpm by magnetic stirrers (Prosense, Oosterhout, The Netherlands). All vessels were at anaerobic conditions by being flushed by N_2_ for 10 min every day.

The fecal slurries were inoculated into colon vessels containing 500 mL (proximal) and 800 mL (distal) of SHIME feed to obtain a final concentration of 1% (*w*/*v*) as described earlier [35]. The SHIME feed (Prodigest, Ghent, Belgium) was prepared according to the manufacturer’s instructions. After inoculation, a steady-state microbial community in the SHIME system was obtained after a stabilization period of 7 days. During the stabilization period, (+)-catechin at a concentration of 200 mg/d was supplemented to proximal colon vessels to avoid the loss of catechin-metabolizing bacteria. In order to stimulate the regular intake of (+)-catechin in humans, a stabilization period was followed by a 21-day treatment period, during which (+)-catechin (200 mg) was administered three times per day to proximal colon vessels. At the end of the SHIME run, a 4-day washout period was included. The samples from this study to characterize catechin metabolism were collected from colon vessels at 0, 2, 4 and 6 h during the last three days of the treatment period (Days 26, 27 and 28) and once per day during the washout period (Days 29, 30, 31 and 32). Obtained samples were then aliquoted for metabolites detection and analysis. All obtained samples were stored at −20 °C immediately after dispensation.

### 3.4. UHPLC-MS Analysis

Samples were pretreated before being applied to HPLC analysis as described previously. Briefly, 1 mL of acidified sample with HCl (70 μL, 7.5% *w*/*v*) was centrifuged at 14,000× *g* for 5 min and filtered by a 0.22 µm membrane filter (Merck, Darmstadt, Germany) [21]. An LC-MS 2020 system (Shimadzu Corporation, Kyoto, Japan) consisting of an electrospray ionization (ESI) source and a single-quadrupole mass analyzer was utilized to quantify metabolites and separate peaks of metabolites. The ESI source was conducted in both positive and negative ion detection mode. Chromatographic separation was conducted on a Phenomenex Luna Omega PS C18 column (2.1 × 100 mm; 1.6 µm particle diameter) at 27 °C. The mobile phase of solvent A was 1% aqueous formic acid, and solvent B was 100% methanol absolute. The flow rate of mobile phases was kept constant at 0.23 mL/min. The gradient elution was set as follows: B from 5 to 40% in 9.5 min; 40% B from 9.5 to 10.7 min; 40 to 100% B from 10.7 to 15.3 min; B from 100 to 5% from 15.3 to 17 min; 5% B from 17 to 17.5 min. The effluent was delivered into a PDA detector (scanning wavelength range, 200–400 nm; resolution, 1.2 nm) and subsequently into an ESI source. The injection volume of samples was 10 µL. The MS parameters were set as follows: *m*/*z* range, 100 to 950; scan speed, 5000 u/s; event time, 0.2 s; nebulizing gas (N_2_) flow rate, 1.5 L/min; drying gas (N_2_) flow rate, 15 L/min; interface temperature, 350 °C; heat block temperature, 400 °C; desolvation line temperature, 250 °C; desolvation line voltage, 0 V; interface voltage, 4.5 kV; Qarray RF voltage, 60 V.

(+)-Catechin and 5-(3′,4′-dihydroxyphenyl)-γ-valerolactone were quantified using a calibration curve prepared with a commercial standard. Calibration curves of standards with eight different concentrations between 0 and 1000 mg/L were prepared, and the correlation coefficient R^2^ was calculated: (+)-catechin (0.9992); 5-(3′,4′-dihydroxyphenyl)-γ-valerolactone (0.9999) (Supplementary Materials Figure S4). The calibration curve of 5-(3′,4′-dihydroxyphenyl)-γ-valerolactone was also used to estimate other metabolites whose commercial standards were not available. The chemical structures of those metabolites without standards were confirmed by following UPLC-IMS-Q-TOF-MS analysis.

### 3.5. UPLC-IMS-Q-TOF-MS Analysis

Intestinal water samples were subjected to ultraperformance liquid chromatography–high-resolution mass spectrometry (UPLC-HRMS) at the VIB Metabolomics Core Ghent (VIB-MCG). A 10 μL volume of sample was injected on a Waters Acquity UHPLC device connected to a Vion HDMS Q-TOF mass spectrometer (Waters, Manchester, UK). Chromatographic separation was carried out on an ACQUITY UPLC BEH C18 (150 × 2.1 mm, 1.7 μm) column (Waters, USA), and column temperature was maintained at 40 °C. Gradient elution of two eluents was used for separation: eluent A (99:1:0.1 water/acetonitrile/formic acid, pH 3) and eluent B (99:1:0.1 acetonitrile/ water/formic acid, pH 3), as follows: 99% A for 0 min decreased to 50% A in 30 min, decreased to 30% from 30 to 35 min and decreased to 0% from 35 to 37 min. The flow rate was set to 0.35 mL/min. ESI was applied, and the LockSpray ion source was operated in negative ionization mode under the following specific conditions: capillary voltage, 3 kV; reference capillary voltage, 3 kV; source temperature, 120 °C; desolvation gas temperature, 550 °C; desolvation gas flow, 800 L/h and cone gas flow, 50 L/h. The collision energy for the full MS scan was set at 6 eV for low energy settings; for high energy settings (HDMSe), it was ramped from 20 to 70 eV. Mass range was set from 50 to 1000 Da, and scan time was set at 0.1 s. Nitrogen (purity > 99.5%) was employed as desolvation and cone gas. Leucine-enkephalin (250 pg/μL solubilized in water/acetonitrile 1:1 (*v*/*v*), with 0.1% formic acid) was used for the lock mass calibration, with scanning every 2 min at a scan time of 0.1 s. Profile data was recorded through Unifi Workstation v2.0 (Waters, Manchester, UK). Data processing was performed with MZmine2 [37].

### 3.6. Data Analysis

Statistical analysis was conducted in GraphPad Prism 9. Indraw (Integle, Shanghai, China) was used to draw the chemical structures in the metabolic pathway.

## 4. Conclusions

This study confirms that (+)-catechin is extensively metabolized by the human microbiota community through batch and SHIME incubation. The results of batch incubation reveal the substantial interindividual variability in the microbial (+)-catechin metabolic efficiency. Based on this variability, the stratification of individuals was conducted to select two individuals for further exploration of metabolic profiles among individuals and colon regions using the SHIME system. Microbial (+)-catechin metabolites, mainly phenylvalerolactones and phenylvaleric acids, were qualified and quantified by UHPLC-MS and UPLC-IMS-Q-TOF-MS. Our results show that various regions of the colon accumulate distinct profiles of microbial (+)-catechin metabolites. Simultaneously, obvious donor-dependent production of these microbial (+)-catechin metabolites was found in the proximal colon rather than the distal colon in terms of both type and amount. However, the individual initial differences in metabolic efficiency of (+)-catechin were not preserved in SHIME, probably due to the adaption period of the simulated colon microbiota to the new conditions. The substantial interindividual variability in microbial metabolism of (+)-catechin and bioaccumulation of metabolites may possess the biological activity and contribute to interindividual differences in the health outcomes of (+)-catechin.

Nevertheless, given that the gut microbiota is the main executor to convert catechin into metabolites with various bioactivities, there is an urgent need to identify the catechin-metabolizing bacteria to further elucidate the interindividual variability in response to (+)-catechin intake.

## Figures and Tables

**Table 1:** Conversion percentage of (+)-catechin in in vitro incubation with fecal microbiota from 12 donors at 4 h. Conversion percentage is expressed as ((Conc._0h_ − Conc._4h_)/Conc._0h_) × 100%. Donors selected for the SHIME run are shown in bold.

Donor No.	Conversion Percentage (%)
**D1**	44.71% (fast)
**D2**	−1.13% (slow) ^1^
D3	35.16%
D4	32.36%
D5	30.15%
D6	3.03%
D7	49.16%
D8	−3.84%
D9	−1.54%
D10	19.66%
D11	36.65%
D12	54.99%

Notes: ^1^ Due to the analytical error of the concentration of (+)-catechin, it was possible that the concentration at 4 h was higher than that of 2 h, resulting in a negative value.

**Table 2:** Identification of potential microbial metabolites based on retention time and MS^2^ fragment profile.

	Chemical	MW (g/mol)	RT (min)	[M-H]^−^ (*m*/*z*)	[MS/MS] (*m*/*z*) Profiles
M1	1-(3′,4′-dihydroxyphenyl)-3-(2′′,4′′,6′′-trihydroxyphenyl) propan-2-ol	292.28	8.28	291.0877	289.07, 123.04, 247.10, 135.04, 167.03, 205.05, 161.06, 151.04, 189.06
M2	5-(3′,4′-dihydroxyphenyl)-γ-valerolactone	208.21	8.35	207.0664	163.08, 123.04, 122.03, 161.06, 164.08, 147.09, 109.02, 153.00, 123.04
M3	4-hydroxy-(3′,4′-dihydroxyphenyl)-valeric acid	226.23	5.98	225.0744	123.04, 163.07, 207.06, 101.02, 223.05, 161.06, 179.07, 122.03, 109.03
M4	5-(3′,4′-dihydroxyphenyl)-valeric acid	210.22	11.84	209.0812	135.05, 191.07, 165.09, 147.08, 163.07, 109.03, 122.04
M5	5-(3′-hydroxyphenyl)-valeric acid	194.23	15.23	193.0888	175.07, 119.05, 147.08, 149.10, 106.04, 99.92, 157,06, 176.08
M6	5-(3′-hydroxyphenyl)-γ-valerolactone	192.21	11.6	191.0699	147.08, 99.92, 106.04, 116.92
M7	5-(3′,4′-dihydroxyphenyl)-4-oxo-valeric acid	224.21	6.63	223.0602	123.04, 179.07, 177.97, 122.03, 161.06, 201.05, 121.03

Notes: MW, molecular weight; RT, retention time.

## Data Availability

The data presented in this study can be provided upon reasonable request.

## Supplementary Materials

Figure S1: Microbial community composition of inoculum of SHIME incubation, Figure S2: Comparison of concentration of (+)-catechin and microbial metabolites between donors and colon region, Figure S3: Incubation of (+)-catechin/Milli-Q water with fecal microbiota from two healthy donors in SHIME system, Figure S4, The standard curve of chemical (a) (+)-catechin and (b) 5-(3′,4′-dihydroxyphenyl)-γ-valerolactone. Table S1: Demographic data of 12 donors enrolled in fed-batch incubation of fecal microbiota with (+)-catechin. Supplementary Method: DNA extraction and library construction, Microbial community analysis.

## Sample Availability

Samples of the compounds are not available from the authors.
