# Supplementary material for: The Donor-Dependent and Colon-Region-Dependent Metabolism of (+)-Catechin by Colonic Microbiota in the Simulator of the Human Intestinal Microbial Ecosystem
Source: Molecules. 2021 Dec 23;27(1):73. doi: 10.3390/molecules27010073 (PMC8746996; doi:10.3390/molecules27010073)
Supplement: Supplementary file 1 [file molecules-27-00073-s001.zip › molecules-1464573-supplementary.pdf]

Supplementary Method

DNA extraction and library construction

500 µL aliquots of fecal slurries were centrifuged (10 min, 5000 *g*) and the supernatant was discarded. The pellet was added 400 mg glass beads (0.1 mm Sartorius, Gottingen, Germany) and 1 ml Lysis buffer (100 mM EDTA, 100 mM Tris, 100 mM NaCl, 1% (w/v) polyvinylpyrrolidone). The samples were put in the PowerLyzer (Mo Bio Laboratories, Carlsbad, USA) at 200 rpm for 5 min, followed by the centrifugation at maximal speed for 5 min, transfer of supernatant to new tubes with 500 µL phenol: chloroform: isoamilyc alcohol 25:24:1 (pH 7) and 700 µL chloroform. The supernatant was obtained after 1 min centrifugation at maximal speed and added with 500 µL phenol: chloroform: isoamilyc alcohol 25:24:1 (pH 7) and 700 µL chloroform. After 1 min centrifugation at maximal speed, 450 supernatant was added with 500 µL ice-cold Isopropyl alcohol and 45 µL 3 M sodium acetate. The resulting solution was cooled at -20°C for 4 h. Then, samples were centrifuged at maximal speed for 1 hour and the DNA was re-suspended by adding 100 µL 1× TE buffer (10 mM Tris, 1 mM EDTA). The library preparation and sequencing were performed on the illumina Miseq platform (Illumina, Hayward, California) by the LGC Genomics (Teddington, Middlesex, UK). V3 to V4 of 16S rRNA gene was amplified with PCR technique with forward primer 341F (5´-CCTACGGGNGGCWGCAG-´) and reverse primer 785R (5´-GACTACHVGGGTATCTAAKCC-3´). The libraries were sequenced on the MiSeq using 2 × 300 paired end mode.

Microbial community analysis

Raw amplicon data was analyzed using DADA2 (Divisive Amplicon Denoising Algorithm) package in R. The primer sequences were firstly cut and reads were truncated at cut-off (truncQ = 2, truncLen= (230, 230)). Sequences were subsequently eliminate ambiguous base calls or reads with errors (maximum expected errors = 2). Then, DADA2 error estimation algorithm and selfConsist sample inference were applied. The denoised reads were combined and the amplicon sequence variant (ASV) table was

obtained based on the resulting error rates. ASV sequences were clustered using Naive Bayesian Classifier and the DADA2 formatted Silva v138. Relative abundances were calculated using Phyloseq package and plotted using ggplot2.

Supplementary Figures

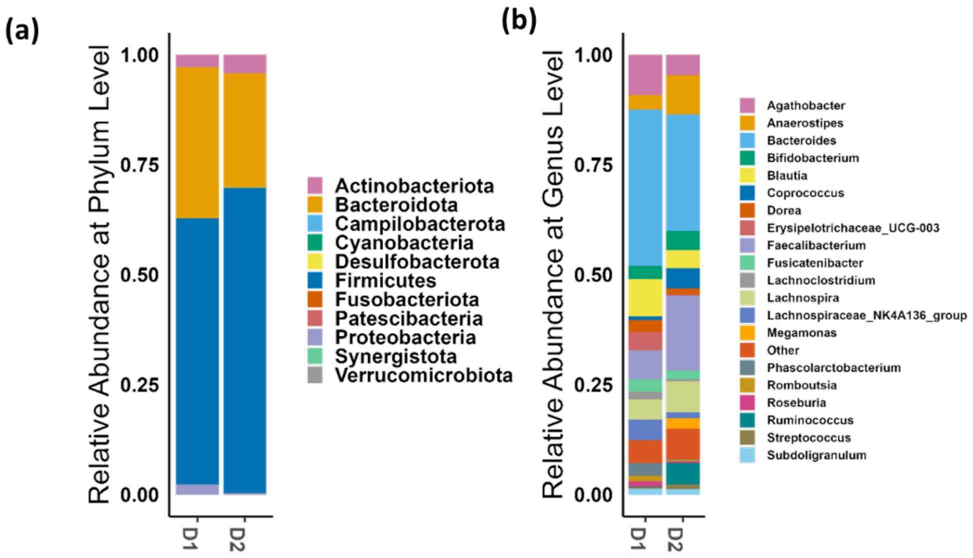

Figure S1. Microbial community composition of inoculum of SHIME incubation. (a) The relative abundance at the phylum level (b), The relative abundance at the genus level constituting > 0.1%. ”Other” is the sum of other genera with relative abundance < 0.1%.

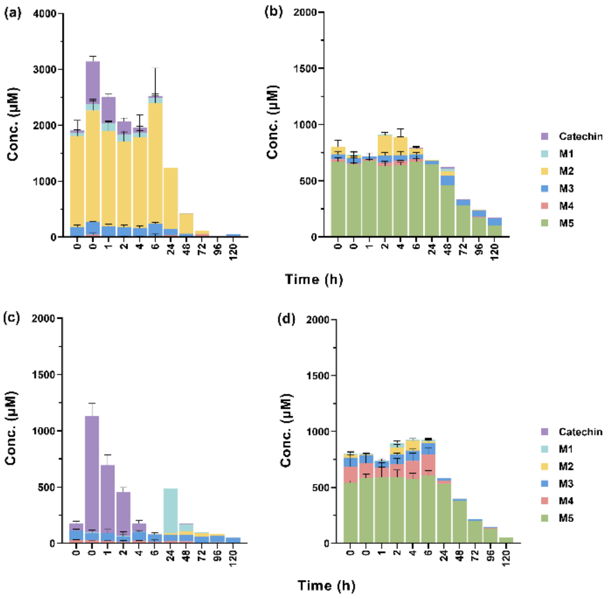

Figure S2. Comparison of concentration of (+)-catechin and microbial metabolites between donors and colon region. (a) Proximal colon vessel of donor 1; (b) Distal colon vessels of donor 1; (c) Proximal colon vessel of donor 2; (d) Distal colon vessels of donor 2. M1, -(3’,4’-dihydroxyphenyl)-3-(2’’,4’’,6’’-trihydroxyphenyl)-2-propanol; M2, 5-(3’,4’-dihydroxyphenyl)-γ-valerolactone; M3, 4-hydroxy-(3’,4’-dihydroxyphenyl)-valeric acid; M4, 5-(3’,4’-dihydroxyphenyl)-valeric acid; M5, 5-(3’-hydroxyphenyl)-valeric acid; M6, 5-(3’-hydroxyphenyl)-γ-valerolactone.

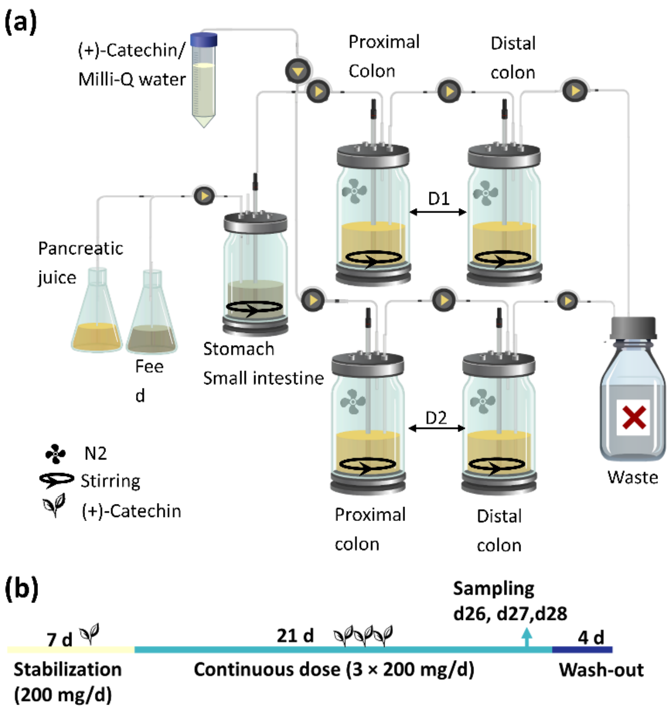

Figure S3. Incubation of (+)-catechin/Milli-Q water with fecal microbiota from two healthy donors in SHIME system. (a) Schematic chart of SHIME system. Each vessel represents a gastrointestinal site; the colon was composed of two distinct vessels to mimic proximal colon and distal colon region. (b) Sampling and polyphenol dosing scheme throughout stabilization and treatment period of the incubation with different supplementation frequency (200 mg/d during stabilization period, 3 × 200 mg/d during the treatment period).

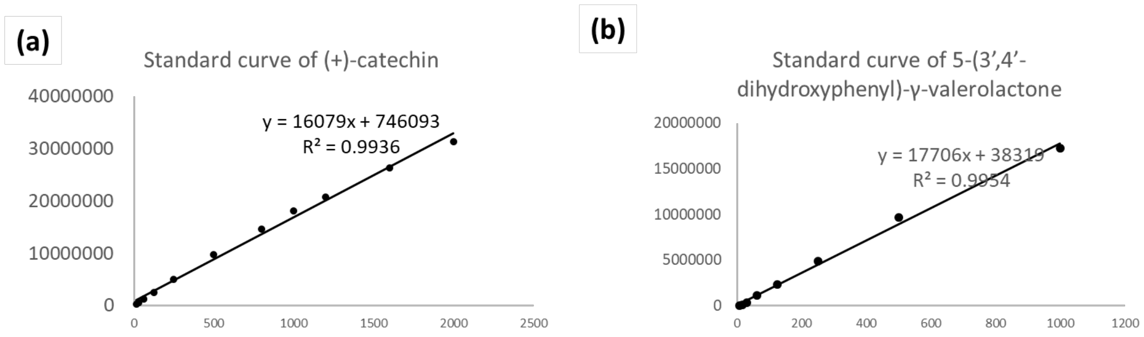

Figure S4. The standard curve of chemical (a) (+)-catechin and (b) 5-(3’,4’-dihydroxyphenyl)-γ-valerolactone. The limit of detection of (+)-catechin: 0.61 mg/L; the limit of quantification of (+)-catechin: 1.37 mg/L; the limit of detection of 5-(3’,4’-dihydroxyphenyl)-γ-valerolactone: 2.38 mg/L; the limit of quantification of 5-(3’,4’-dihydroxyphenyl)-γ-valerolactone: 7.93 mg/L.

Table S1 Demographic data of 12 donors enrolled in fed-batch incubation of fecal microbiota with (+)-catechin

Donor No.	Gender[1]	Age (year)	BMI (kg/m^2)
D1	F	27	20.98 (fast)
D2	F	30	23.88 (slow)
D3	F	27	21.94
D4	F	26	21.80
D5	M	28	19.72
D6	M	26	25.90
D7	M	27	27.76
D8	F	30	20.06
D9	M	25	20.45
D10	F	26	17.69
D11	M	27	23.66
D12	F	28	21.30

Notes: [1] M, male; F, female.
